# Supplementary material for: Study to Determine the Prevalence of Menstrual Migraine in Reproductive-Age Women in Saudi Arabia
Source: Healthcare (Basel). 2024 Jan 25;12(3):317. doi: 10.3390/healthcare12030317 (PMC10855712; doi:10.3390/healthcare12030317)
Supplement: Supplementary file 1 [file healthcare-12-00317-s001.zip › healthcare-2769966-supplementary.pdf]

We are a research group from the College of Medicine at King Khalid University conducting a study titled 'The Relationship between Premenstrual Syndrome and Menstrual Migraine.' Participation in this study is voluntary, and you can choose not to participate if you wish. Participation involves filling out a questionnaire through the attached link, taking approximately five minutes. Your answers will be confidential, and no identifying information will be collected. The results of this study will be used for academic purposes only, and there is no harm in participating in this study at all.

Do you currently use any type of contraception?
- Yes
- No

Have you been previously diagnosed with ovarian cysts?
- Yes
- No

Have you undergone ovarian removal surgery?
- Yes
- No

Have you ever experienced headaches?
- Yes
- No

When you have a headache, does it last from 4 hours to three days (when not using any treatment)?
- Yes
- No

When you have a headache, which of the following symptoms occur with it? (Choose all symptoms you experience)

- Intense headache on one side of the head.
- Headache worsens with movement.
- Pulsing around the eyes and head.
- Nausea or vomiting.

When you have a headache, how often is the pain severe?
- Never
- Rarely
- Sometimes
- Often
- Always

How much does a headache limit your ability to perform daily activities, including household chores, work, school, or social activities?
- Never
- Rarely
- Sometimes
- Often
- Always

When you have a headache, how often do you wish you could lie down?
- Never
- Rarely
- Sometimes
- Often
- Always

In the past four weeks, how often did you feel severe fatigue from performing work or daily activities due to headaches?
- Never
- Rarely
- Sometimes
- Often
- Always

In the past four weeks, how often did you feel frustration or anger due to headaches?
- Never
- Rarely
- Sometimes
- Often
- Always

In the past four weeks, how often did headaches limit your ability to concentrate on work or daily activities?
- Never
- Rarely
- Sometimes
- Often
- Always

How many days in the past three months did you miss work or school due to headaches? (Specify zero if it didn't happen)

How many days in the past three months did your productivity at work or school decrease by half or more due to headaches? (Do not include days counted in the previous question where you missed work or school.)

How many days in the past three months did you avoid household tasks (such as housework, repairs and maintenance, shopping, child and family care) due to headaches?

How many days in the past three months did your productivity in household work decrease by half or more due to headaches? (Do not include days counted in the previous question where you didn't do household chores.)

How many days in the past three months did you miss family, social, or recreational activities due to headaches?

One week before your menstrual cycle, do you experience any of the following symptoms? (Choose all symptoms you experience)

- Noticeable emotional sensitivity (e.g., mood swings, sudden feelings of sadness or crying, or increased sensitivity to rejection).
- Increased or noticeable anger, irritability, or escalating personal conflicts.
- Noticeably depressed mood, feelings of despair, or thoughts of self-disapproval.
- Anxiety, tension, and/or feelings of tightness or stress.
- Reduced interest in usual activities.
- Difficulty concentrating.
- Lethargy, easy fatigue, or a noticeable decrease in energy.
- A significant change in appetite. Overeating or a strong desire to eat.
- Excessive sleep or insomnia.
- Feeling confused or out of control.
- Physical symptoms such as breast pain or swelling, joint or muscle pain, feeling "bloated," or weight gain.
